# Improving hospital safety for patients with chronic kidney disease: a mixed methods study

**DOI:** 10.1186/s12882-021-02499-4

**Published:** 2021-09-23

**Authors:** Lucia New, Donna Goodridge, Joanne Kappel, Joshua Lawson, Roy Dobson, Erika Penz, Gary Groot, John Gjevre

**Affiliations:** 1 Health Sciences Program, College of Medicine, University of Saskatchewan, Saskatoon, SK Canada; 2 Department of Medicine, College of Medicine, University of Saskatchewan, 103 Hospital Drive, SK S7N OW8 Saskatoon, Canada; 3 Department of Medicine, Saskatchewan Health Authority, Saskatoon, Saskatchewan Canada; 4 Department of Medicine, University of Saskatchewan, Saskatoon, Saskatchewan Canada; 5 College of Pharmacy and Nutrition, University of Saskatchewan, Saskatoon, SK Canada

**Keywords:** Chronic kidney disease, Communication, Emotional safety, Mixed methods research, Patient feedback, Patient safety

## Abstract

**Background:**

People living with chronic kidney disease (CKD) require complex medical management and may be frequently hospitalized. Patient safety incidents during hospitalization can result in serious complications which may negatively affect health outcomes. There has been limited examination of how these patients perceive their own safety.

**Objectives:**

This study compared the safety perceptions of patients hospitalized with CKD using two approaches: (a) the Patient Measure of Safety (PMOS) questionnaire and (b) qualitative interviews. The study objectives were to: (1) assess concordance between qualitative and quantitative data on safety perceptions and (2) better understand safety as perceived by study participants.

**Methods:**

A cross-sectional convergent mixed methods design was used. Integration at the reporting level occurred by weaving together patient narratives and survey domains through the use of a joint display. Interview data were merged with results of the PMOS on a case-by-case basis for analysis to assess for concordance or discordance between these approaches to safety data collection.

**Results:**

Of the 30 inpatients with CKD, almost one quarter (23.3 %) of participants reported low levels of perceived safety in hospitals. Four major themes emerged from the interviews: receiving safe care; expecting to be taken care of; expecting to be cared for; and reporting safety concerns. Suboptimal communication, delays in care and concerns about technical aspects of care were common to both forms of data collection. Concordance was noted between qualitative and quantitative data with respect to communication/teamwork, respect and dignity, staff roles, and ward type/lay-out. While interviews allowed for participants to share specific concerns related to safety about quality of interpersonal interactions, use of the questionnaire alone did not capture this concern.

**Conclusions:**

Safety issues are a concern for in-patients with CKD. Both quantitative and qualitative approaches provided important and complementary insights into these issues. Narratives were mostly concordant with questionnaire scores. Findings from this mixed methods study suggest that communication, interpersonal interactions, and delays in care were more concerning for participants than technical aspects of care. Eliciting the concerns of people with CKD in a systematic fashion, either through interviews or a survey, ensures that hospital safety improvement efforts focus on issues important to patients.

## Background

CKD is a global health issue with a world-wide prevalence of 9.1 % [1]. In Canada, the number of people on chronic dialysis nearly doubled between 2000 and 2019 [2]. The complexity of this disease and its medical management predispose individuals to further complications of the cardiovascular and renal systems [3, 4]. Under-recognition of impaired kidney functioning [5–9], presence of multiple comorbid conditions [3, 5, 10], and polypharmacy associated with management of comorbidities [6] often culminate in frequent hospital admissions. In a population of pre-dialysis patients with CKD in the Safe Kidney Care (SKC) Cohort Study, Ginsberg et al. [4] reported that 70 % experienced at least one adverse safety incident or actionable safety finding, while over one-third had two or more. Self-reported safety incidents from this same study included self-reported hypoglycemia and falls, as well as incidentally detected hypotension and hypokalemia [11]. Safety incidents have the potential to further compromise renal functioning and accelerate the progression of kidney disease, contributing to morbidity and mortality [8]. Since hospitalization rates are higher for patients with kidney disease [12], increasing awareness and taking measures to avoid safety incidents are key to protecting this patient population from harms. Their experiences with safety are an important contribution to the patient safety literature.

The safety of patients remains a top priority for healthcare organizations as evidenced by safety practices that are currently implemented in various healthcare settings, focusing on incidents related to medication errors, hospital acquired infections (HAI), and medical/clinical procedures [13–16]. In contrast, safety concerns perceived by patients tend to be non-clinical in nature and associated with emotional and psychological impact [17], including communication amongst health care providers, information shared by providers, and relational aspects of care [18–23] Integrating the patient lens into safety improvement strategies allows for care processes and issues that are important to patients to be addressed [24], helping to inform safety strategies for addressing psychological and emotional as well as physical harms.

The shift towards patient-centered care in health care has helped to advance the measurement and integration of patient feedback in quality and safety improvement priorities. Patient reported outcome measures (PROMs) and patient reported experience measures (PREMs) are standardized and validated instruments to be completed by patients. PROMs demonstrate patients’ perceptions regarding the effectiveness of clinical treatment and health related quality of life whereas PREMs look at the process of care and the impact on patients’ experiences [25–27]. The goal of this study was to explore CKD patients’ perceptions of safe care and therefore, patient experiences were deemed to be more relevant than patient reported outcomes. Patient experience data have the potential to identify perceived problems with care delivery, inform continuous improvement of services, evaluation of service redesigns [25] and have been positively associated with patient safety [28]. Surveys and questionnaires are commonly used but patient experience data have also been obtained through qualitative interviews [29]. Feedback simultaneously obtained using surveys and patient narratives may provide contrasting or complementary information to inform safety and care improvement strategies. In a study conducted by Tsianakas and colleagues [27], similar issues were identified using a survey instrument and patient interviews although the two methods differed in aspects of care emphasized.

The purpose of this mixed methods study was to explore CKD patients’ perceptions of safety during their most recent hospitalization using the Patient Measure of Safety (PMOS) questionnaire [30] and face-to-face interviews. As a patient reported measurement tool, the PMOS survey items ask about aspects of care that patients are able to provide feedback on, such as communication, coordination of care, and staff availability. By having patients identify risks to their safety based on factors and processes within the care environment, hospitals can use this data to assist with safety improvements. The study objectives were to: (1) assess concordance between qualitative and quantitative data on safety perceptions and (2) better understand safety as perceived by the participants in this study through a mixed methodology approach.

## Methods

### Study design

A cross-sectional convergent mixed method design (QUAL + Quan) [31–33] was used to examine safety within acute care hospital environments as perceived by patients with CKD. Qualitative and quantitative data were collected concurrently. The two sets of data were merged to determine confirmation or discordance once the interview responses and questionnaire results were analyzed separately.

### Setting and sample

Patients living with any stage of CKD, hospitalized for health problems other than specifically for dialysis treatments, were recruited from medical and surgical inpatient care units at one urban tertiary hospital in Western Canada between October 2017 and February 2018. Each unit had a mix of private and shared rooms with patient census at maximum capacity most days. Due to the limited availability of private rooms, it was not uncommon for these patients to be in a room with individuals on infection precautions. Although a system was in place for staff, patients, and families to report safety concerns by phone, this access was not visible nor communicated to patients.

Purposive sampling was used to recruit participants as the primary objective of this study was to examine CKD patients’ perspectives on safety while in hospital. By attending weekly interdisciplinary nephrology patient care rounds, the researcher was able to obtain the names of eligible individuals who might be interested in being part of the study. Patients were personally recruited by the researcher to participate in the study if they were: (a) over 18 years of age at CKD stages 3, 4 or 5 (ESRD); (b) currently admitted for reasons other than dialysis treatment; (c) able to provide informed consent and (d) able to participate in face-to-face interviews and complete a questionnaire. Patients who were non-English speaking were also eligible if an interpreter was available.

### Data collection

All data were collected at participants’ bedsides during their current hospital admission. Interviews were conducted first, followed by completion of the Patient Measure of Safety (PMOS) questionnaire [30]. Patient characteristics including age, sex, education level, stage of CKD, length of time living with CKD, and the number of times hospitalized in the last five years were collected before the start of the interviews. The length of time for both sets of data collected ranged between 30 and 90 min.

### Qualitative data

Participant narratives were obtained using a semi-structured interview guide [21]. To set the context for patient safety, each interview opened by asking participants to recall personal experiences with safety incidents or whether family and friends had incurred harm while in hospital. Participants were then asked about their current hospitalization with the opportunity to answer in a non-structured format. Additional questions were asked as required to prompt or clarify responses. With permission from participants, interview responses were audio recorded.

### Quantitative data

Following the interview, participants were asked to fill out the PMOS questionnaire. It took between 10 and 15 min for each questionnaire to be completed. The PMOS instrument was developed based on findings from a research project where factors frequently contributing to errors experienced by participants were identified and finalized through a consensus decision-making process that led to the development of the PMOS in its first iteration, the version used in the current study [30, 34]. A study to validate the PMOS was conducted at one acute teaching hospital in England in which 297 patients or family members participated [34]. Pearson’s correlation (r = 0.79, p = 0.007, k = 10) was used to determine convergent validity of the questionnaire while discriminant validity was determined using a multivariate analysis of variance (MANOVA) procedure (Wilk’s λ = 1.67, df = 110, 1510, *p* < 0.001). Test-retest reliability was also determined using Pearson’s correlation (r = 0.75 for overall PMOS score).

Forty-three Likert-style items comprised the questionnaire, from strongly disagree (1) to strongly agree (5). One item specifically asked about perceptions of being treated with dignity and respect. The remainder of the items were categorized into domains of patient safety including communication and teamwork (9 items), organization and care planning (5 items), access to resources (3 items), ward type and layout (11 items), information flow (3 items), staff roles and responsibilities (4 items), staff training (2 items), and equipment design and functioning (3 items). Higher overall PMOS scores reflect a higher level of safety perceived.

### Data analysis

#### Qualitative

Audio recorded interview data were professionally transcribed verbatim. Transcriptions were checked against audio recording and entered into QSR NVivo® Version 11 for coding. Initial analysis of participant responses informed subsequent interview sessions. Emerging patterns were further explored in successive interviews. Once interviews concluded, data were analyzed to identify patterns and themes following the process outlined by Braun and Clarke [35]. By reading through the data set repeatedly, initial codes were generated. Once the list was finalized, codes were sorted into themes and subthemes. Themes were then reviewed in relation to the research objective of understanding safety as perceived by study participants.

#### Quantitative

Analysis of quantitative survey data was performed using SPSS (version 25). For each participant, the overall PMOS score as well as individual scores for each domain of safety were calculated. The scores were directly related to the level of safety perceived. Descriptive analysis of the overall PMOS score was performed and quartile values were used to categorize participants into groups of low (below 25th percentile; PMOS score < 15.5), moderate (between 25th and 75th percentile; PMOS score ≥ 15.5, ≤ 29.24), or high (above 75th percentile; PMOS > 29.24) safety level perceived. Data analysis consisted of comparisons between participant characteristics and overall PMOS scores. Chi square analysis was performed when using the categorized PMOS scores both categorically and continuously. To confirm the results from the categorical analysis, further analysis using the continuous PMOS score was conducted using the Mann Whitney test. Spearman’s rho was used to determine the correlation between the individual domains of safety, the question item regarding dignity and respect, and the overall PMOS score. Statistical significance for bivariate analysis was set a priori at p < 0.05. A detailed discussion on the quantitative analysis is reported in another article [36].

### Data integration

Integration at the reporting level occurred by weaving together patient narratives and PMOS domains using a joint display [31, 37]. Interview data were merged with results of the PMOS questionnaire for participants on a case-by-case basis for analysis. A matrix table was created to perform an initial comparison of qualitative and quantitative results for each participant to determine whether responses related to the domains of safety were present. Quantitative and qualitative data were presented side-by-side to determine concordance or discordance, whether qualitative responses were neutral or if there was silence from either the narratives or the PMOS questionnaire. Concordance was determined to occur when interview data were congruent with domain ratings (e.g., when coding for that section of the individual participant’s qualitative data reflected a positive appraisal and the individual domain score was also high in this area). Divergence occurred if the two sets of data differed, allowing for a more thorough examination of factors contributing to the specific domains of safety. Qualitative responses were considered neutral if they neither confirmed nor contradicted questionnaire results. Silence resulted from the absence of qualitative responses corresponding to the domain ratings or the lack of quantitative data matching narratives from participants.

### Ethics

 Ethics approval was granted by the affiliated University Behavioural Ethics Board (17–300) as well as the Institutional Research Ethics Board (16–309).

## Results

Participant characteristics.

Thirty individuals living with CKD participated in this study. One additional patient was approached and declined but did not provide a reason. Slightly over half were 65 years old or younger. Approximately one-third of the respondents have had kidney disease for six years or longer. Many of the participants had ESRD as well as two or more comorbid conditions. Three quarters of participants were on hemo- or peritoneal dialysis. Twelve participants had been in the hospital more than 10 times in the past five years. Detailed demographic and clinical characteristics are available in Table 1.

**Table 1:** Participant Characteristics (*n*=30)

Characteristics	n (%)
Sex	
Male	16 (53.3)
Female	14 (46.7)
Age	
≤ 65 years	17 (56.7)
≥ 66 years	13 (43.3)
Self-declared Ethnicity	
Non-Caucasian Canadian	16 (53.3)
Caucasian Canadian	14 (46.7)
Education Level	
< Gr. 9	7 (23.3)
Grades 10-12	20 (66.7)
Post-secondary	3 (10)
Admitting Diagnosis	
Infection of extremities	9 (30.0)
Infection to dialysis catheters	3 (10.0)
Other (ie. cardiac, pneumonia, infection (other), amputation)	18 (60.0)
Current Length of Stay	
≤ 15 days	21 (70.0)
≥ 16 days	9 (30.0)
Number of hospitalizations in past 5 years	
<5 times	12 (40.0)
5-10 times	6 (20.0)
>10 times	12 (40.0)
Comorbidities	
Present (diabetes, hypertension, PVD)	25 (83.3)
Absent	5 (16.7)
Length of CKD	
≤ 5 years	19 (63.3)
≥ 6 years	11 (36.7)
Stage of CKD	
3	3 (10)
4	4 (13.3)
5 (ESRD)	23 (76.7)
Renal replacement therapy or conservative treatment	
Hemodialysis	17 (56.7)
Peritoneal	6 (20.0)
Currently not on dialysis	7 (23.3)

Table reproduced with permission from CANNT Journal (2020), Volume 30, Issue 1

### Quantitative Findings

Descriptive analyses were performed on the overall PMOS and individual domain scores. Participants were categorized into low, moderate, and high groups using the percentile values of the overall PMOS score. Seven participants (23.3 %) had overall PMOS scores falling within the low (< 15.5) perceived levels of safety, 16 (53.3 %) had scores within the moderate (15.5-29.24) perceived levels, and scores for seven individuals (23.3 %) were at high (> 29.24) perceived levels of overall safety. There was no statistical significance between participant characteristics and overall PMOS score. Strong or moderate direct correlations were found between each PMOS domain and the overall PMOS score. Quantitative findings have been published elsewhere [36].

### Qualitative Findings

 In recounting their current and in some cases most recent past hospital experiences, study participants brought up issues associated with technical/clinical and interpersonal aspects of care. Four main themes and 13 subthemes emerged from analysis of interview responses. Three themes were related to the perceptions of care and personal safety and included: *receiving safe care; expecting to be taken care of; and expecting to be cared for*. The fourth theme, *reporting safety concerns*, pertained to the willingness of participants to be involved in their own safety through speaking up or informing providers. Table 2 lists the themes and subthemes.

There were participants who implicitly trusted their health care providers and expressed that the care received was as expected but there were others who felt that care could be improved. For participants who spoke up at the point of care, this generally involved asking questions or seeking clarification from staff. Speaking up was different than reporting incidents or concerns. There was a general sense of hesitancy with reporting for reasons of disbelief that any significant change would occur and apprehension regarding the impact on care. A more detailed report was previously published [21].

**Table 2:** Themes and subthemes from participant responses

Themes	Subthemes
Receiving safe care	Sharing a room with patients on isolation Roommates perceived to be threatening Lack of cleanliness Other patients and visitors
Expecting to be taken care *of*	Communication amongst providers Communication between providers and participants Delays in care
Expecting to be cared *for*	Interactions with health care providers Trust
Reporting safety concerns	Speaking up Reactions to questions asked Awareness of safety line Using the safety line

### Integration of Qualitative and Quantitative Data

In addition to the overall PMOS score, participants were also categorized into low, moderate, or high score groupings for each of the domains. Table 3 displays the number of participants and corresponding percentages for the three categories of the different domains. Using joint displays, comparisons of interview data with their PMOS domain scores were reviewed for each of the participants in their respective categories.

**Table 3:** Percentages of participants per low, moderate or high PMOS score categories

	Participant Categories						
Domains & Ratings	Low score group (n=7)	percentage of group (%)	Moderate score group (n=16)	percentage of group (%)	High score group (n=7)	percentage of group (%)	Percentage of total participants (%)
Respect & dignity							
Low	4	57.14	4	25.00	2	28.57	33.33
Moderate	2	28.57	7	43.75	2	28.57	36.67
High	1	14.29	5	31.25	3	42.86	30.00
Communications, Teamwork							
Low	6	85.71	1	6.25	0	0.00	23.33
Moderate	1	14.29	11	68.75	2	28.57	46.67
High	0	0.00	4	25.00	5	71.43	30.00
Organization, Care planning							
Low	5	71.43	2	12.50	0	0.00	23.33
Moderate	2	28.57	9	56.25	3	42.86	46.67
High	0	0.00	5	31.25	4	57.14	30.00
Access to resources							
Low	5	71.43	2	12.50	0	0.00	23.33
Moderate	2	28.57	9	56.25	3	42.86	46.67
High	0	0.00	5	31.25	4	57.14	30.00
Ward type & layout							
Low	5	71.43	2	12.50	0	0.00	23.33
Moderate	2	28.57	12	75.00	0	0.00	46.67
High	0	0.00	2	12.50	7	100.	30.00
Information flow							
Low	4	57.14	6	37.50	1	14.29	36.67
Moderate	3	42.86	7	43.75	0	0.00	33.33
High	0	0.00	3	18.75	6	85.71	30.00
Staff roles							
Low	5	71.43	4	25.00	1	14.29	33.33
Moderate	1	14.29	5	31.25	1	14.29	23.33
High	1	14.29	7	43.75	5	71.43	43.33
Staff training							
Low	1	14.29	5	31.25	1	14.29	23.33
Moderate	6	85.71	5	31.25	2	28.57	43.33
High	0	0.00	6	37.50	4	57.14	33.33
Equipment design & function							
Low	6	85.71	5	31.25	1	14.29	40.00
Moderate	1	14.29	7	43.75	3	42.86	36.67
High	0	0.00	4	25.00	3	42.86	23.33

Tables 4 and 5 present the joint display of the mixed methods integration. For the domains that were commented on by participants, interview responses were mostly concordant with the scoring, regardless of whether the domains were rated low, moderate, or high. More detailed responses were provided by participants in the low and moderate categories. Safety perceptions were based solely on quantitative data for domains that were absent of participant narratives. Domains related to information flow and equipment design were two such examples. Participants also brought up perspectives that were not explored by the PMOS questionnaire, such as the importance of family members being involved in the care and professional conduct of staff.

**Table 4:** Joint display of quantitative, qualitative, and mixed methods meta-inferences: Communication and interpersonal aspect of care

PMOS Domain (25th, 75th percentiles)	Concordance	Discordance	Neutral	Silent
Respect & dignity (2, 5)	I hope the old elderly people don’t get treated like that…Talking to me like I was senile or something like that… Talk to me like I’m you Domain rating: 2 (Grant)	They are much more caring and much more doing of small things really well [citing example of fixing the clock so he can tell when meals come, bed time, etc] Domain rating: 2 (Henry)	Because they treat me good Just be kind to the patients Domain rating: 5 (Nellie)	Domain rating: 1; no interview data to support. (Ulrich)
Communication & teamwork (26.5, 35)	As for the nephrology department you get bounced around from doctor to doctor to doctor. I don’t even know who my doctor is and they change stuff all the time Domain rating: 23 (Edward)	I think they can give me more information, you know, because a lot of times you are moving here and doing things and you don’t ask, next thing it’s too late and they change nurses all the time and switching shifts Domain rating: 32 (Kevin)	No interview data to support	Domain rating:24; no interview data to support. (Evan)
Organization & care planning (14.8, 19)	So now you feel like you’re in lock up. Nobody’ll tell you what’s going on. Nobody will come see you. Domain rating: 14 (Evan)	…but a doctor should be aware and nurses should communicate…I think communication throughout the system is kind of hard on patients. Domain rating: 18 (Kip)	No interview data to support	Domain rating:19; no interview data to support. (Amsel)
Staff roles (11, 14)	She said I’ll help you so I was hoping she’d wash my back at least, cause I can’t do it and she just walked away and she didn’t help me nothing. She didn’t do nothing for me Domain rating: 11 (Una)	Do their jobs properly, if they are not doing their jobs properly how do you expect to get good care and everything else, I don’t know any other way how to word that Domain rating: 14 (Boyd)	No interview data to support	Domain rating: 11; no interview data to support. (Brenda)

**Table 5:** Joint display of quantitative, qualitative, and mixed methods meta-inferences: Technical aspects of care

PMOS Domain (25th, 75th percentiles)	Concordance	Discordance	Neutral	Silent
Access to resources (11.8, 15))	They need to get more staff it’s just that simple - it seemed like to me they’re short on staff all the time Domain rating: 8 (Lisa)	And I kept telling, I said I need a bedpan. …Well the next thing was me and the bed were soaking wet. And they got annoyed. Domain rating: 16 (Frances)	No interview data to support	Domain rating: 10; no interview data to support. (Barbara)
Ward layout (27.8, 37.3)	Especially when there’s like with people with the gowns, the diseases or something. I yeah didn’t feel safe when I was in a room with them. Domain rating: 23 (Kip)	I’m not showering here cause like the shower was like filthy dirty Domain rating: 35 (Debbie)	Bigger rooms, better things I guess Domain rating: 23 (Betty)	Domain rating: 26; no interview data to support. (Lisa)
Staff training (4.8, 8)	Some are mistakes but that was lack of knowledge Domain rating: 4 (Ulrich)	No, I don’t say anything. They all do stuff different I noticed. Domain rating: 9 (Betty)	… the nurses come and help me and they take care of me the best way they know how. Domain rating: 8 (Grant)	Domain rating: 4; no interview data to support. (Mariette)

### Respect and dignity

Perceptions of negative interactions with staff were described primarily by participants who provided low ratings for this domain, indicating concordance with the domain ratings. Conversely, respondents with high scores discussed how they felt cared about as a person. There were also neutral responses such as expressing the wish “to have nurses who cared” or “you get to know the nurses who show you respect and dignity”. A few respondents shared comments that appeared to contradict the ratings assigned to the domain. For example, one participant had expressed appreciation for the promptness of care and assistance and yet rated this domain to be low.

### Communication and teamwork

Most of the interview responses were concordant with the ratings with the majority of the narratives from participants with low and moderate ratings for this domain. Concerns were expressed about the lack of communication among care providers, causing participants to feel anxious about inconsistencies of care. Participants with low scores for this domain expressed frustration with the lack of updated information provided, while those with moderate ratings felt that the information shared met their needs without overwhelming them.

### Organization and care planning

 A mixture of concordant and discordant responses was observed from participants in the low and moderate categories respectively. Concerns regarding the number of health providers involved in their care were expressed. Respondents perceived that treatment plans appeared to be dependent on the physician on service for that week and spoke of inconsistent information shared by staff and clinicians. A few participants with moderate ratings described experiences that were incongruent with the score. For example, one participant voiced surprise and appreciation for the way a physician explained an upcoming procedure to enhance his understanding. Another participant expressed apprehension when describing perceptions of disorganized care by the healthcare team.

### Staff roles

A majority of interview responses were concordant with the PMOS data and were mostly related to staff responsibilities rather than to roles specifically. Narratives by participants with low scores were related to deficiencies in care whereas for respondents who rated this domain to be high, perceptions of care met expectations. There were also a few comments discordant with quantitative data, such as comments related to nursing staff being afraid to do more or staff to “just do their job properly”, both from participants who had rated this domain to be high.

### Access to resources

Unlike the previous domains, responses were fewer in number and primarily from participants in the moderate category. Narratives were predominantly related to perceived staffing shortages or workload. Frustration was expressed with delays in care and responses to call bells but there were also expressions of sympathy towards the nursing staff.

### Ward layout

The two main issues raised by participants with low and moderate ratings were the lack of cleanliness and sharing a room with another patient with an infectious condition. Regardless of whether participants rated this domain to be low or moderate, similar concerns were shared about hospital acquired conditions from either the substandard hygienic condition of the environment or the infectious nature of their roommates.

### Staff training

Interview responses were mostly concordant with the PMOS data with discussions around clinical practices of nursing staff over that of any other care provider group. Respondents with low or moderate ratings brought up examples of nurses being rough or non-compliant with infection control practices. Participants with high scores expressed significant regard for the competency of nursing staff and believed this provider group knew the patients best, therefore engendering significant degree of trust. Neutral comments included ones where participants indicated they were not in a position to judge the competency of care providers.

## Discussion

A comparison of the data from individual interviews and the PMOS questionnaire yielded similarities and differences between the data sets. Narratives were mostly concordant with questionnaire scores, regardless of whether ratings for the safety domains were low, moderate, or high. Findings from this mixed methods study suggest that communication, interpersonal interactions, and delays in care may be more concerning than technical aspects of care for respondents. The patient safety movement is about preventing harms associated with the process of healthcare and while substantial progress has been made in protecting patients from physical or physiological harms, reducing emotional and psychological harms was as significant for participants in this study.

With the many examples of narratives concordant with the low or low-moderate ratings for the communication/teamwork and organization/care planning domains, it was clear that communication amongst health care providers was a significant concern for participants. Communication can be challenging with the number of care providers involved in patients’ care with information regarding patients’ plan of care inadequately shared, leading to inconsistent or missed care. The inconsistent or lack of communication among providers meant that participants had to be vigilant about their care, which was not possible for, nor wanted by, all respondents. More concerning is that over the course of a patient’s hospital stay, missed communication can have a cumulative effect, eventually resulting in harm to the patient [38].

 Furthermore, some participants expressed frustration with the communication between providers and themselves. The lack of information shared with participants may have the effect of silencing their voices, reinforcing the passive role of the patient, and conveying a lack of respect for their knowledge or their need for information. When information is not shared, patients may find it difficult to ask questions or participate in their care and safety. Lowey and colleagues [39] reported that patients are apprehensive about the loss of control and worry about deteriorating health during hospitalizations. For some of the participants who were already dependent on technology and health care providers to maintain their health, being involved in their own care by having information shared with them was important as it enabled them to feel safe and in control of their situation.

Discordance was also noted between some of the domain ratings and the interview data. Possible reasons for the discrepancies may be due to how participants answered based on their interpretation of survey items, perceptions of situations that prompted the response, and influence from previous experiences. Discordant findings were useful to illuminate additional factors considered by participants to be important for their safety while hospitalized, a finding supported by Huppertz and Smith [40] who maintained that by providing context and concrete situations, patient concerns can assist in safety improvement efforts. Although ratings for the respect and dignity, communication/teamwork, and organization/care planning domains were high for some participants, the presence of family to advocate for the patient was an essential element of feeling safe in the hospital. Standards of patient care should include discussing with patients the involvement of family members in care planning.

 Interview responses such as “to have nurses who care” or “to be treated fair and well” may indicate that the participant has had experiences to the contrary but was unwilling to discuss it during the interview. Participants were also silent on many of the domains, possibly because the topic or question may not be important to them at that point in time, or participants were unwilling to speak up or share with the researcher, especially while they were still in the hospital. Regardless of whether comments were concordant, discordant, neutral, or absent, combining narrative and questionnaire responses enabled a deeper understanding of safe care as perceived by study participants.

Using the PMOS to obtain feedback as well as interviewing participants was more informative than if the questionnaire had been the sole method utilized. Triangulation of the quantitative and qualitative data allowed a more complete picture of patient safety to emerge. While the PMOS was a useful metric to monitor safety from the perspective of this patient population, issues flagged in the interviews did not completely fit with the domains of safety being evaluated. Without the interviews, hospital care experiences that participants deemed to be important, that either negatively or positively impacted on their overall perceptions of safe and compassionate care, would not have been disclosed. Burt and colleagues [41] support the value of using both quantitative and qualitative assessments, noting that “literal interpretations of absolute scores may overstate quality of care if not considered in a fuller context” (p. 7).

For study participants, interpersonal aspects of health care such as attitudes of care providers and communication with providers assumed as much importance as clinical competence toward their perceptions of safety. Narratives from participants indicated concerns for their emotional safety, which was influenced by interpersonal relations and interactions with their care providers, as well as access to timely care and information. Although participants were able to identify perceived levels of safety related to technical/clinical aspects of care, numerous interview responses included negative interpersonal interactions that left participants feeling uncared for and therefore unsafe. Other studies have similarly identified the loss of respect and dignity from negative interpersonal interactions with providers [41–45], leading patients to feel psychologically and emotionally unsafe [43, 45], particularly for ones with high recurrence of hospital care.

Communication was another important factor contributing to participants’ perception of safety. Deficient communication amongst multiple care providers made participants question the care received and/or required them to be vigilant which was taxing for some respondents. Issues with communication suggested family presence was important for participants to feel safe. Communication has been used as a way of maintaining the power differential inherent in health care[46, 47, 48]. Power is exerted through what and how much is communicated as well as the words and language that are used[46]. This power must be shared to encourage patients who are willing and capable to be involved in care decisions and maintaining their safety, with the goal of achieving better health outcomes.

The responsibility for maintaining the safety of patients is borne primarily by the health care system despite calls for the engagement of patients and families in this process. While measures taken by the health care system are critical for maintaining the safety of patients, study findings indicated that participants also took actions to protect themselves from HAIs and medication errors but were vulnerable to emotional and psychological harms. Some participants seemed prepared to take on a more active role if they had been encouraged. The concept of patient safety is most often described as the reduction or absence of preventable harm associated with the provision of health care. If this is indeed the goal, then engaging with patients who are willing and able to participate in safety improvement has the advantage of ensuring that strategies encompass the priorities of patients as well as the health care system, addressing physical, psychological, and emotional harms.

## Strengths and Limitations

Data were collected while participants were hospitalized, reducing the risk of recall bias. The researcher was careful to avoid the use of leading interview questions to prevent biasing participants’ responses. A previously validated instrument [34] to assess patients’ perceptions was used for the quantitative method, while 30 interviews were analyzed using thematic analysis. In the process of data analysis, the researcher remained open to all possible themes to reduce confirmation bias. Integration of the data followed the mixed methods approach outlined by Fetters [32] and allowed for the direct comparison for two distinct approaches to evaluating safety perceptions.

This study was conducted at a single institution which is one of two provincial referral sites for kidney disease. While the sample size of 30 participants was appropriate for this novel comparison of qualitative and quantitative safety perception data, this sample size limits the generalizability. Responses to the questionnaire may be influenced by fatigue since completion was done following the interview, which lasted 45 min or longer for some participants. Social bias may have affected how participants answered some of the questions. Nonetheless, the combination of questionnaire data and interview responses enabled a more descriptive picture of participants’ perceptions of safety. The positive and negative experiences shared by participants illustrate the value of including patients in safety improvement.

## Future Research

Participants were either in CKD stage 4 or 5 (ESRD). Comparing this group with individuals at CKD stages 1 or 2 may be interesting to ascertain any differences in safety perceptions. Additional research involving a larger sample size with patients with other health conditions will further inform safety improvement initiatives. Inclusion of patients who experience language barriers will also be beneficial. In using the PMOS for future studies, asking participants to provide rationale for their ratings may further enhance understanding of perceptions of safety specific to the domains and overall safety. Nevertheless, the process of the current study and results were instructive in learning about safety from the perspectives of patients with CKD. In sharing their experiences, participants provided insights into what may be considered safe care for patients.

## Conclusions

The focus on patient-centered care and safety movement has provided an impetus to obtain patient feedback regarding their hospital care experience. Participants living with CKD were recruited for this mixed methods study to gain an understanding of their perceptions of safety during an acute care hospital admission with feedback obtained through interviews and the PMOS questionnaire. Concordance between participant narratives and PMOS domains expanded on quantitative ratings. Both concordant and discordant findings, as well as additional interview responses, enabled a contextualized understanding of hospital experience of care and safety from the participants in this study. While there were domain ratings where participant narratives were absent, they did show that patients are able to determine safety risks from these factors. Safety priorities for participants included quality interpersonal interactions, access to timely care and information, and communication. By examining safety as perceived by patients with CKD, findings from this study have helped to advance the patient safety movement by demonstrating that some patients are able to partner with health care systems to develop safety improvement plans. Including patients with CKD in safety improvement will ensure care that is safe, reliable, and effective from the perspectives of both patients and health care organizations, thereby protecting this higher risk population.

## Data Availability

Datasets generated and analyzed are not publicly available in order to protect the identity of participants. De-identified information may be available from the corresponding author on reasonable request.

## Abbreviations

CKD: Chronic kidney disease
ESRD: End stage renal disease
PMOS: Patient Measure of Safety
